# Enhanced Inhibition of Amyloid Formation by Heat Shock Protein 90 Immobilized on Nanoparticles

**DOI:** 10.1021/acschemneuro.3c00370

**Published:** 2023-07-20

**Authors:** Ana Rodríguez-Ramos, Jesús A. González, Mónica L. Fanarraga

**Affiliations:** Grupo de Nanomedicina, Universidad de Cantabria, Instituto Valdecilla - IDIVAL, Avda. Herrera Oria s/n, Santander 39011, Spain

**Keywords:** molecular chaperone, protein renaturation, amyloid, neurodegeneration, nanorobot, microrobot

## Abstract

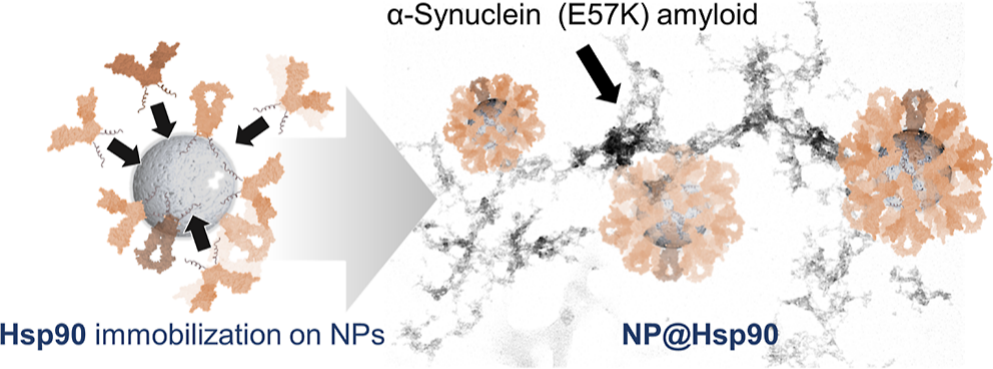

As the population ages, an epidemic of neurodegenerative diseases with devastating social consequences is looming. To address the pathologies leading to amyloid-related dementia, novel therapeutic strategies must be developed for the treatment or prevention of neural protein-folding disorders. Nanotechnology will be crucial to this scenario, especially in the design of nanoscale systems carrying therapeutic compounds that can navigate the nervous system and identify amyloid to treat it in situ. In this line, we have recently designed a highly simplified and versatile nanorobot consisting of a protein coating based on the heat shock protein 90 (Hsp90) chaperone that not only propels nanoparticles using ATP but also endows them with the extraordinary ability to fold and restore the activity of heat-denatured proteins. Here, we assess the effectiveness of these nanosystems in inhibiting/reducing the aggregation of amyloidogenic proteins. Using Raman spectroscopy, we qualitatively and quantitatively analyze amyloid by identifying and semi-quantifying the Amide I band. Our findings indicate that the coupling of Hsp90 to nanoparticles results in a more potent inhibition of amyloid formation when compared to the soluble protein. We propose that this enhanced performance may be attributed to enhanced release–capture cycles of amyloid precursor oligomers by Hsp90 molecules nearby on the nanosurface. Intelligent biocompatible coatings, like the one described here, that enhance the diffusivity and self-propulsion of nanoparticles while enabling them to carry out critical functions such as environmental scanning, identification, and amyloid prevention, present an exceptional opportunity for the development of advanced nanodevices in biomedical applications. This approach, which combined active biomolecules with synthetic materials, is poised to reveal remarkable prospects in the field of nanomedicine and biotechnology.

## Introduction

The problem of protein misfolding and aggregation underlies many neurodegenerative diseases, including those with distinct genetic makeups, clinical symptoms, and prevalence, including Alzheimer’s, Parkinson’s, Huntington’s disease, or amyotrophic lateral sclerosis.^1^ In all of these syndromes, the accumulation of abnormal proteinaceous material in the form of amyloid is a key event in the pathological cascade. Amyloid deposits unchain a series of degenerative processes that leads to neuronal dysfunction and cell death. Unfortunately, despite significant advances in the understanding of the mechanisms of many protein-conformational diseases, there are still no treatments to prevent or eliminate amyloid. Given the nature of these disorders, a crucial therapeutic approach would involve stabilizing the intermediate conformations of amyloidogenic proteins (such as β-amyloid, Tau, α-synuclein, SOD1, TDP-43, and so forth) to impede their aggregation.^2,3^ Regrettably, the options for pharmacological treatments are currently quite limited. One example is the utilization of chemical chaperones, which can be used to hinder or assist in the process of refolding misfolded proteins.^4^

In nature, the problem of protein folding is predominant. For this reason, a large part of our genome encodes protein genes whose only function is to interact with unfolded or aggregated polypeptides to stabilize or refold these or promote their destruction. Some of these “refolding” proteins belong to the family of molecular chaperones and can antagonize amyloid toxicity by modulating protein aggregation pathways.^5^ As a result, boosting the folding capacity of tissues, for example, by locally increasing the amount of chaperones that block the development or promote the breakdown of amyloid, could be a feasible therapeutic approach to prevent accumulative neurodegenerative pathologies.

The Hsp (heat shock protein) family, which falls under the category of molecular chaperones, encompasses a group of proteins that are universally present and have significant involvement in numerous physiological processes related to the folding and refolding of molecules especially when responding to various types of stress. Some of these are involved in amyloidogenic diseases such as Alzheimer’s disease,^6−8^ Parkinson’s disease,^9,10^ amyotrophic lateral sclerosis (ALS),^11,12^ or polyglutamine disorders.^13,14^ In fact, the majority of research suggests that Hsp proteins play a neuroprotective function by diminishing the amounts of misfolded proteins both in vitro and in vivo, mitigating the buildup of amyloid aggregates and the associated pathological outcomes.^15,16^

Among the various Hsp chaperones available, we selected Hsp90 for our study due to several compelling reasons. Foremost, this chaperone exhibits exceptional versatility, capable of binding to and repairing a diverse range of client polypeptides. Moreover, Hsp90 plays a vital role in numerous essential physiological processes, further emphasizing its significance in our research.^17^ Second, both in vitro and in vivo, Hsp90 is a crucial chaperone in the control and suppression of the intermediate phases of amyloid formation.^7,16,18−20^ This chaperone is also present in amyloid plaques^21^ and bound to α-synuclein (α-Syn) in the Lewy bodies in the brains of Parkinson’s patients^22^ where it appears to inactivate the harmful amyloidogenic oligomers.^19,20,23^ Based on these results, we expect that, as with enzymes^24,25^ and other immobilized chaperones,^26−28^ we will be able to enhance the anti-amyloidogenic activity of Hsp90 recruited to the surface of nanoparticles.

**Figure 1:**
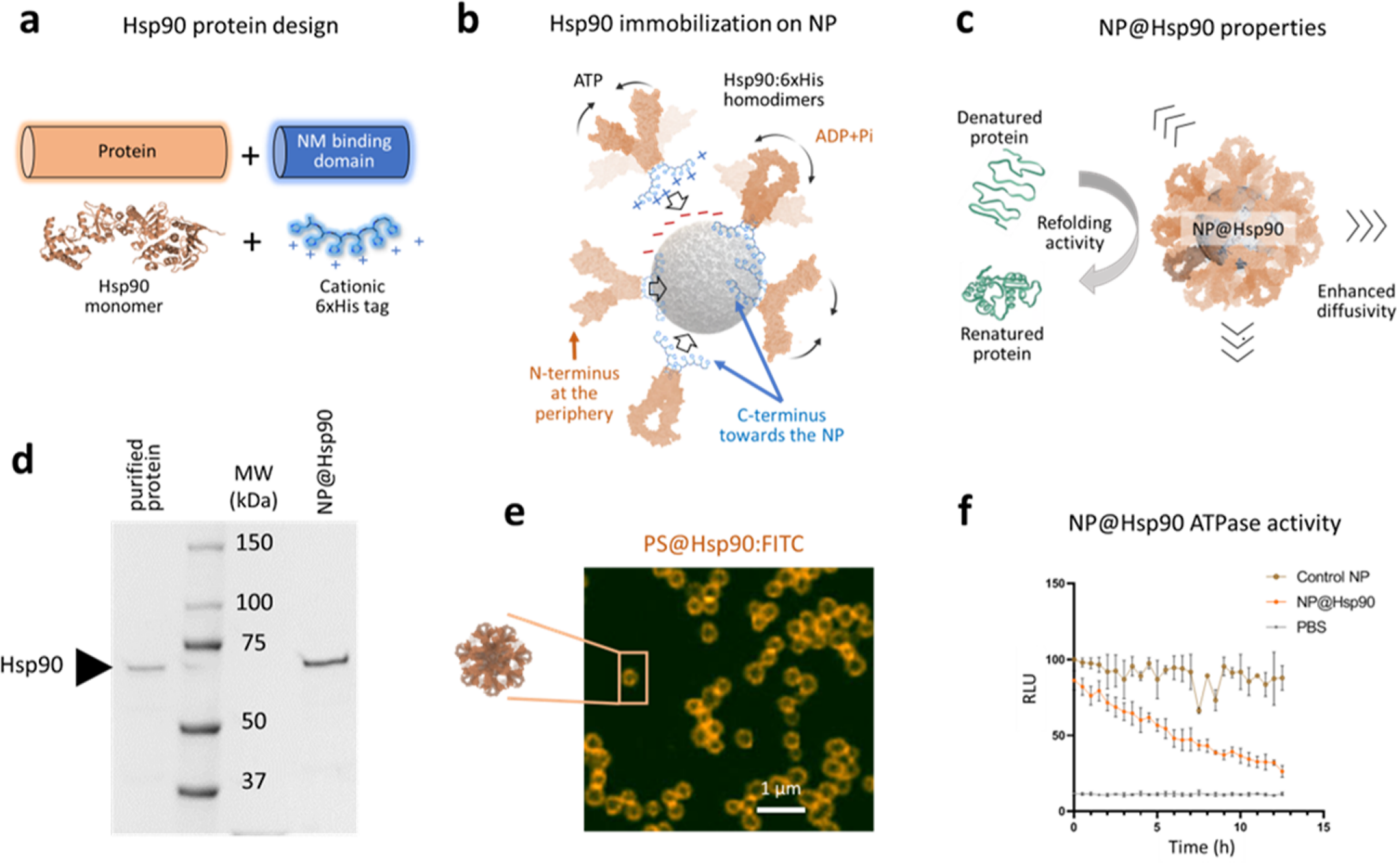
NP@Hsp90 design. (a) Diagram of the engineered Hsp90:6xHis protein. The nanomaterial (NM) binding domain of the recombinant protein is a cationic peptide (6xHis) bound to the C-terminus of Hsp90 (blue). (b) Diagram of the assembly of the immobilization of Hsp90 on nanoparticles (NP). The NM binding domain is electrostatically recruited on the negatively charged nanoparticles correctly positioning the protein. Black arrows indicate the opening-closing motions of the Hsp90 homodimer during the ATP hydrolysis cycle. (c) Diagram of the NP@Hsp90 multifunctionality. (d) SDS-PAGE gel image showing the purified Hsp90:6xHis band (left lane), molecular weight (MW) markers, and Hsp90 detached from functionalized nanoparticles (right lane). (e) Confocal microscopy image of a single *Z*-plane of polystyrene (PS) particles coated with Hsp90:6xHis:FITC. The fluorescent halo corresponds to the FITC-stained Hsp90. (f) Quantitative luminescent ATP detection assay to evaluate the ATPase activity of Hsp90 after immobilization on the PS particles. The graph shows the amount of ATP as a function of light, expressed in RLU (see the Experimental Section). The activity of Hsp90 bound to the PS particles results in a decrease of ATP in the medium which, in turn, results in a reduction of the luminescence. Naked particles are used as a negative control.

Of particular interest, Hsp90, functioning as an ATPase, undergoes structural modifications through ATP hydrolysis, enabling it to capture and repair proteins. Our recent research reveals that nanoparticles of different types, when incorporating immobilized Hsp90 on their surfaces, can self-propel. This transformation effectively turns these nanosystems into “nanoswimmers” that can repair proteins that have been denatured by heat within their proximity (Figure 1).^29^ Thus, this study aims to test the hypothesis that enhancing the anti-amyloidogenic activity of Hsp90 recruited to nanoparticle surfaces (NP@Hsp90) could potentially lead to the development of novel nanotherapies that can navigate the nervous system via ATP hydrolysis, recognizing and refolding amyloid on the fly.

## Results and Discussion

### Hsp90 Immobilization on the Particle

Consistent with prior research, Hsp90 was attached and immobilized on NPs by modifying the protein sequence using genetic engineering. The *htpG* GENE ID:945099 (Methods, Figure S1) was used to create a fusion protein that was modified in the 3′ region to provide the final protein a cationic hexahistidine peptide sequence at the carboxyl terminus to enable electrostatic attachment in a predetermined position to negatively charged nanomaterials (ζ-potential of −20 or lower) (Figure 1a–c). Previous studies have demonstrated that electrostatic binding can be utilized effectively to bind various synthetic proteins and nanomaterials. This binding interaction exhibits remarkable stability under physiological conditions, including a wide pH range of 5.2–9.0, high salt concentrations of up to 1 M NaCl in the medium, and even elevated temperatures of up to 90 °C for durations exceeding 15 min^29,30^ Furthermore, we have shown that the genetic changes we have introduced on Hsp90 allow for its correct positioning on the nanosurface and do not affect its ability to hydrolyze ATP or fold/repair other proteins.^29^

Using the aforementioned versatile binding methodology, we achieved successful immobilization of Hsp90 on two different types of particles, serving as proof of concept for this approach. We employed carboxylate-modified polystyrene (PS) spheres of ca. 500 nm diameter, which displayed a ζ-potential value of ca. −50 mV (PS@Hsp90), and silica (SiO_2_) nanoparticles of approximately 100 nm diameter with a ζ-potential value of ca. −20 mV (SiO_2_@Hsp90). The functionalization of the particles was biochemically demonstrated using SDS-PAGE electrophoresis and confocal microscopy (Figure 1d,e respectively). The total amount of protein bonded to the surface of the particles was estimated in ca. 0.2 μg/mg of recombinant protein of PS particles, and 0.3 μg/mg for the SiO_2_ nanoparticles. The functionality of the immobilized recombinant Hsp90 was evaluated using a quantitative luminescent ATP detection assay kit (abcam ref. ab113849) (Figure 1f) as described in the Experimental Section.

### Evaluation of Free and Immobilized Hsp90’s Anti-Amyloidogenic Activity

To investigate the anti-amyloidogenic activity of NPs@Hsp90 in vitro, we synthesized a form of the amyloidogenic human α-Syn protein, the α-Syn-E57K mutant. This particular protein is known to aggregate in the central nervous system of individuals with Parkinson’s disease.^31,32^ Its identification in 1997 revealed its role as one of the primary contributors to the complex neurodegenerative disorder, which affects a substantial population of over 10 million individuals worldwide.

**Figure 2:**
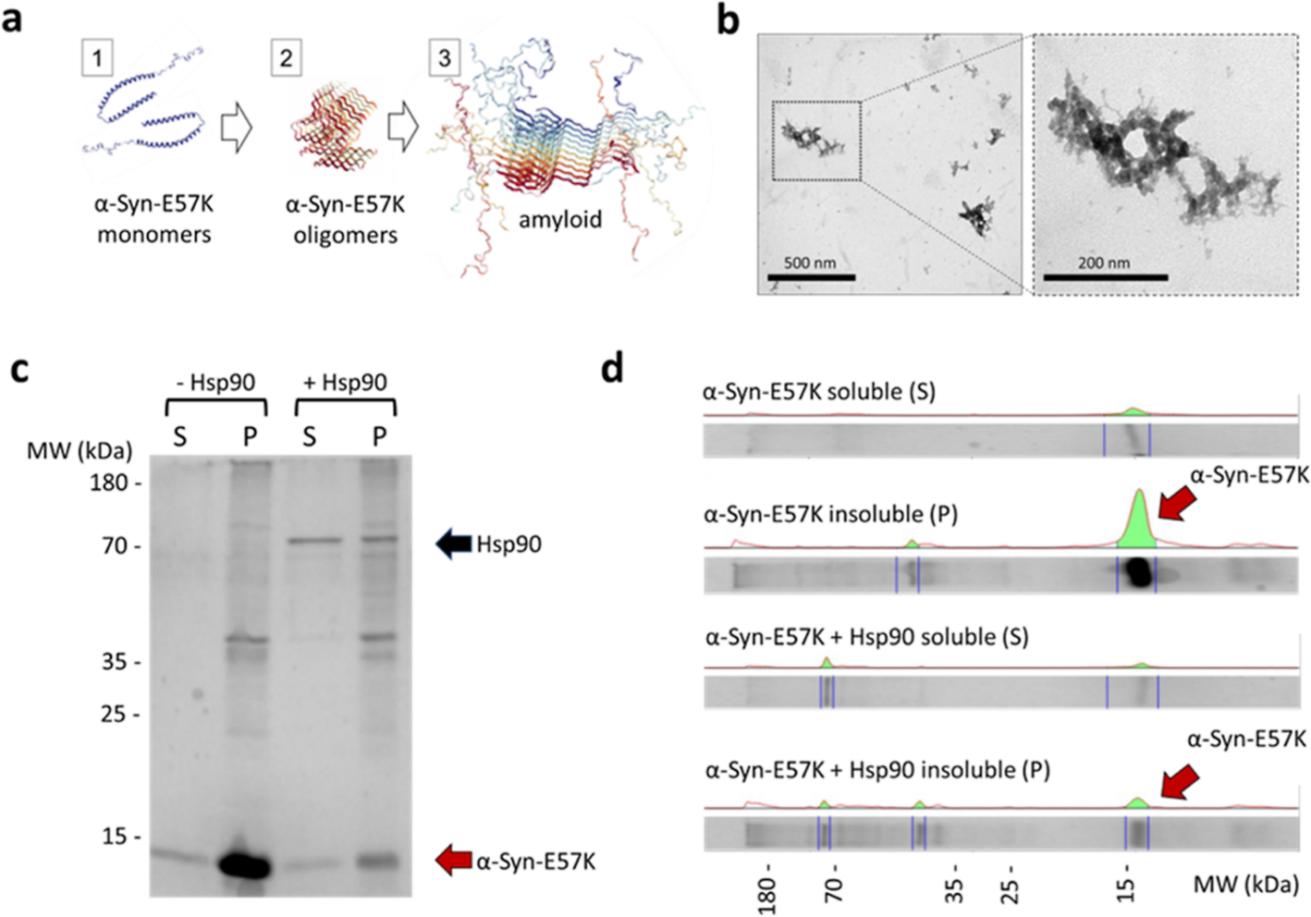
In vitro amyloid formation. (a) Diagram of human mutant α-Syn-E57K protein: #1 monomers, #2 oligomers, and #3 amyloid fibers (structures from PDB database refs. 1XQ8, 6A6B, and 2N0A, respectively). (b) Representative TEM images of the in-house produced α-Syn-E57K oligomers. (c) Analysis of α-Syn-E57K aggregation after incubation under amyloidogenic condition in the presence/absence of Hsp90. To analyze the samples, a centrifugation step was performed to separate the soluble protein fraction (S) from the insoluble protein aggregates (P). The separated fractions were then subjected to analysis using SDS-PAGE. (d) Representative protein landscape profiles for each of the lanes of the gel in (c) obtained with ImageLab. In the presence of Hsp90, the total amount of insoluble α-Syn-E57K oligomers decrease significantly.

Under amyloidogenic conditions (72 h incubation at 37 °C),^32^ the mutant α-Syn-E57K protein naturally assembled amyloid oligomers in vitro (Figure 2a). Figure 2b shows a transmission electron microscopy (TEM) image of the amyloid oligomers assembled in vitro using the in-house α-Syn-E57K produced protein. To validate the anti-amyloid properties of the engineered Hsp90, a biochemical study was performed. The α-Syn-E57K samples were exposed to amyloidogenic conditions, with and without the chaperone. Following this, the soluble and aggregate protein fractions were separated and run in SDS:PAGE gels to investigate the impact of the chaperone on amyloid formation. Figure 2c,d demonstrate the anti-amyloidogenic activity of the dispersed (free) form of Hsp90. This is more visible in the protein landscape representation depicted in Figure 2d, where the red arrows highlight the impact of Hsp90 in mitigating amyloid formation visible in the insoluble protein fraction. Quantitative analysis of the protein bands confirmed a decrease in the insoluble fractions from 18.42 to 5.67% in the presence of Hsp90. These findings confirm the functionality of the modified Hsp90 and demonstrate the significant role that dispersed Hsp90 plays in preventing the accumulation of α-Syn-E57K.

Next, we proceeded to assess the effects of dispersed Hsp90 chaperone in comparison to two nanosystems, each carrying the protein on the surfaces of distinct cores. One nanosystem involved polystyrene particles (PS@Hsp90), while the other employed silica nanoparticles (SiO_2_@Hsp90). For the test, equal samples of human α-Syn-E57K protein were incubated with identical amounts (0.2 μg/μL) of Hsp90 protein either dispersed in the medium or bound to the respective particles for the comparative analysis. These samples were exposed to amyloidogenic conditions for 72 h at 37 °C with rotation.

When assessing the amyloid content in the sample, we encountered a lack of a definitive and precise method in the existing literature for performing qualitative and quantitative analysis of amyloid fibers oligomeric precursors (Figure 2A). Amyloid is often measured using dyes that have an affinity for fully formed amyloid fibers detected in nerve tissue accumulated over time. However, it is important to note that the affinity of these dyes for oligomeric amyloid precursors, which tend to form during shorter incubation periods, is not consistently reliable.^33,34^ Furthermore, accurately detecting and quantifying these oligomeric precursors through biochemical methods is a significant challenge.

### Raman Spectroscopy Demonstrates NPs@Hsp90 Efficiently Reduce Amyloid Oligomerization

Considering the limitations associated with traditional methods for detecting amyloid oligomers, we opted to employ Raman spectroscopy as a means to reliably identify the presence of β-sheet structures, which are characteristic of amyloid formation. This approach allowed us to unambiguously confirm the presence of amyloid in samples subjected to aggregation conditions and also provided a semi-quantitative estimation of the amount of amyloid present in the samples.^35−37^

In line with existing literature, we analyzed the intensity of the Amide I peak (1665 cm^–1^) that is characteristic of the β-sheet conformation of the amyloid oligomers. To semi-quantify the intensity of the Amide I peak and evaluate the aggregation level of the α-Syn-E57K protein, we used the intensity of the CH_3_–CH_2_ strain band (at 1451 cm^–1^) as a normalization factor for the obtained spectra.

**Figure 3:**
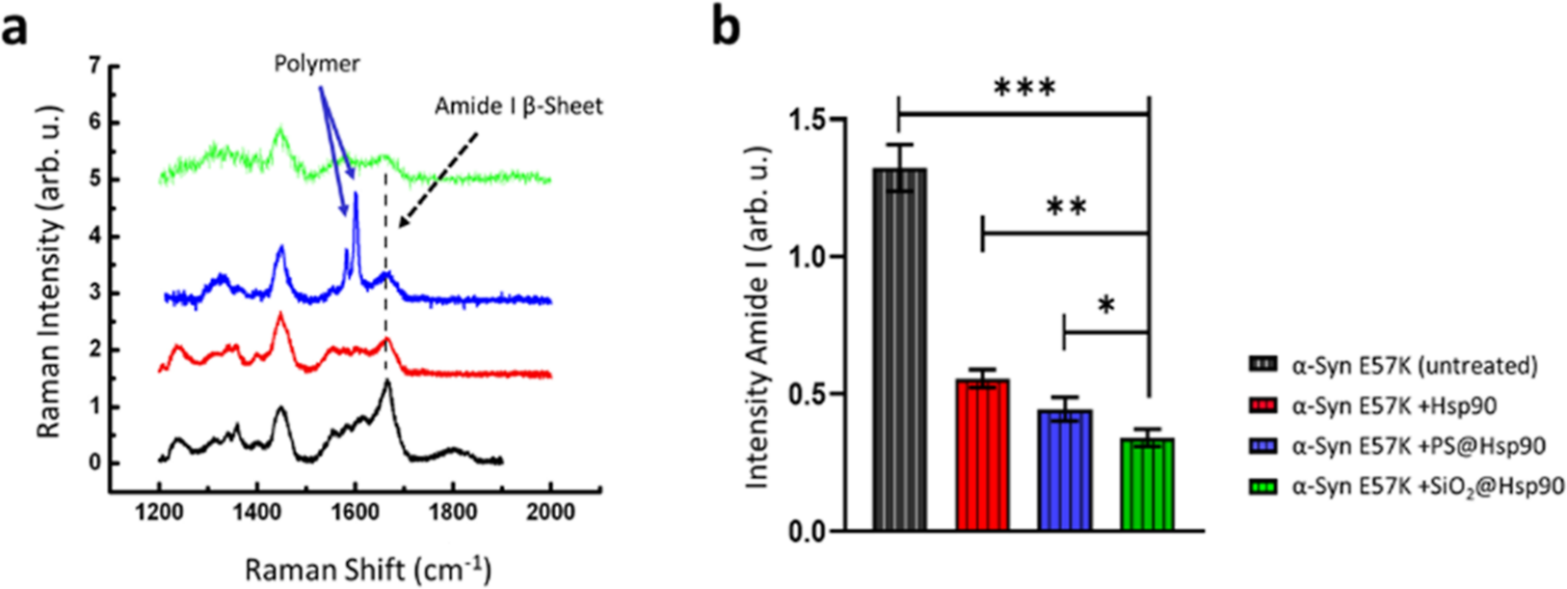
Evaluation of the anti-amyloidogenic effect of the Hsp90 coating. (a) Normalized Raman spectra obtained from samples of human α-Syn E57K mutant protein incubated under amyloidogenic conditions. Measurements on the untreated control (black), the sample containing free Hsp90 chaperone dispersed in the medium (red), sample treated with PS@Hsp90 nanoparticles (blue, polystyrene-specific peaks are indicated by arrows), or the SiO_2_@Hsp90 particles (green). In all samples containing Hsp90, a decrease in the intensity of the amide I band representative of α-Syn amyloid oligomers is observed (vertical dashed line). The decrease in the Amide I peak is more significant in the two samples containing the chaperone bound to the particles. (b) Semiquantitative representation of the Amide I β-sheet peak intensity after spectra normalization (Table S1). The anti-amyloidogenic effect was more intense when Hsp90 was immobilized on NPs. Values for the two-tailed Student’s *t*-test are indicated with asterisks (*p*-values: (*) = 0,0267; (**) = 0,0012; (***) <0,001; *n* = 3).

For the analysis, we conducted three replicate Raman spectroscopic measurements on samples derived from three separate aggregation experiments. Raman signals were collected following the procedure outlined in the experimental section. In Figure 3a, the normalized Raman spectra clearly demonstrate a noticeable decrease in the intensity of the Amide I band (indicated by the dashed line) in all samples that were subjected to incubation with Hsp90. Both the samples treated with free Hsp90 and immobilized Hsp90 exhibited a reduction in the intensity of the band.

Semiquantitative evaluation of the intensity in the Amide I β-sheet peaks (represented in Figure 3b and detailed in Table S1) exhibited a statistically significant reduction in all samples when the Hsp90 protein was present. Notably, this reduction became more pronounced when Hsp90 was bound to particles and particularly more prominent when it was immobilized onto particles at the nanoscale. SiO_2_@Hsp90 nanoparticles of 100 nm show significantly better efficacy in preventing α-Syn-E57K aggregation (*p* < 0.001; *n* = 3) than larger PS particles of approximately 500 nm in diameter. This suggests that the increased surface area facilitates a higher binding capacity and enhances the anti-amyloidogenic activity of the immobilized chaperone, leading to improved inhibition of amyloid formation.

## Conclusions

In this study, we investigated the ability of free Hsp90 chaperone and Hsp90-coated nanoparticles to prevent the formation of amyloid fiber precursors formed by the α-Syn-E57K protein associated with Parkinson’s disease. Our findings confirm the anti-amyloidogenic properties of the engineered Hsp90 protein and support the hypothesis that its effectiveness is enhanced when immobilized on particles. Through Raman spectroscopy analysis, we observed a significant improvement in this effect when Hsp90 was immobilized on particles at the nanoscale. These results highlight the potential of Hsp90-based nanotherapies for mitigating amyloid formation and providing valuable insights for future research in this area.

These findings suggest two potential mechanisms through which the immobilized chaperone enhances the anti-amyloidogenic effect. First, the presence of multiple neighboring Hsp90 molecules on the surface of nanoparticles can facilitate the rapid capture of oligomers released from one chaperone to another in close proximity. This amyloid folding effect reduces the time it takes to capture the oligomer, resulting in a more efficient anti-amyloidogenic process. This mechanism can be compared to a “chain folding reaction” that accelerates the prevention of amyloid formation. Second, the structural changes in the Hsp90 dimer, which serves as the propulsion system of the nanoparticles,^29^ can contribute to the improved performance of the system. These changes enhance the diffusivity of the particles, particularly in the smaller ones, leading to more effective recognition, capture, and folding reactions of the substrate.

In summary, this study successfully demonstrates the potential of designing nanoscale anti-amyloidogenic systems by utilizing a protein coating, specifically Hsp90, that can effectively capture and refold amyloid oligomers. The incorporation of Hsp90 coating also provides the nanosystems with self-propulsion capabilities, allowing them to navigate nerve tissue and prevent amyloid formation on-the-fly.

Furthermore, the versatility of the Hsp90 protein coating enables its assembly on various types of nanomaterials, expanding its potential applications. This adaptable coating holds promise for further improvements, such as optimizing core size or incorporating it into drug nanocarriers, thereby enhancing the anti-amyloid effects of therapeutic agents. Overall, these characteristics make it a highly promising candidate for biomedical applications in the field of neurodegenerative diseases.

## Methods

### Gene Synthesis

Synthetic chimera recombinant 6xHis fusion gene constructs encoding the bacterial Hsp90 chaperone (*htpG* GENE ID:945099) and the amyloidogenic mutant α-synuclein (SNCA-E57K, NCBI GENE ID:6622) were both synthesized and cloned in the bacterial vector pET 15b plasmid system (Novagen) by General Biosystems, Inc. (Morrisville, USA).^38^ Following the cloning of these genes, full plasmid sequencing was performed to verify the cloned sequences. The final recombinant-protein sequences of Hsp90 and the mutant SYN-E57K proteins are detailed in Figure S1. The plasmids were transformed into the *E. coli* DH5α bacterial strain for their conservation, and the *E. coli* BL21 strain for protein expression.

### Protein Expression and Purification

One Shot BL21 (DE3) *E. coli* (ref. EC0114, Thermo Fisher Scientific) cells were transformed with the expression vectors using standard methods. For protein expression, bacterial cultures were grown in Luria–Bertani (LB) broth supplemented with antibiotics (100 μg/mL ampicillin) until *A*_600_ ca. 0.6. Protein expression was induced by adding 0.5 mM isopropyl b-d thiogalactopyranoside. All proteins were produced and purified in our laboratories following standard biochemical procedures. After protein expression, bacterial pellets were resuspended in 50 mM NaH_2_PO_4_, 300 mM NaCl, pH 8.0 with protease inhibitor (Pierce, Thermo Fischer Scientific). Clarified cell lysates were obtained by probe sonication followed by a centrifugation cycle. The proteins were purified in pre-equilibrated Ni-TED columns (Protino Ni-TED, Macherey-Nagel) and finally passed through PD-10 Columns (GE Healthcare) to remove the imidazole. Biochemical protein analysis was performed using SDS-PAGE electrophoresis (Figure 1d). Coommassie-stained gels were digitalized and analyzed using the BioRad GelDoc EZ system software. Hsp90 labeling with fluorescein isothiocyanate (FITC, Sigma-Aldrich) was done as previously described.^39^ The protein solution in phosphate-buffered saline (PBS) was first treated with 1 M sodium bicarbonate buffer, pH 8.8, adding 0.1 mL per 1 mL of protein. Then 50 μL of 5 mg/mL FITC solution in DMSO were slowly added under continuous stirring, and the reaction was kept for 1 h at room temperature. The labeled protein was separated from the unconjugated FITC using gel filtration (Sephadex G-25 resin) PD-10 columns (GE Healthcare, Chicago, IL, USA).

### Nanoparticle Coating with Hsp90

SiO_2_ nanoparticles of 100 nm diameter (Sigma-Aldrich 797936-5MG) and colorless carboxylate-modified latex beads (Polysciences, Inc. Ref. 09836) of 500 nm diameter were functionalized with saturating amounts of the 6xHis-tagged purified Hsp90 resuspended in PBS using mild sonication as previously described.^30,39−41^ In brief, ca. 100 μg of particles were immersed in 500 μL PBS containing saturating amounts of the purified tagged protein (ca. 0.2 mg/mL) at room temperature. The mixture was sonicated in a water bath for 5 min. Unbound protein was removed by repeated centrifuge washes. SDS-PAGE electrophoresis was used to quantify the protein captured on the surfaces of the particles (Figure 1b). The attached protein was stripped in Laemmli sample buffer (BioRad) at 90 °C and was loaded in SDS-PAGE acrylamide gel for electrophoresis (Figure 1d). Semi-quantification of the protein on the particles was performed on Coomasie-stained gels using the software of the BioRad GelDoc EZ system. Approximately 0.4 μg of recombinant protein was captured per mg of the particles.

### Validation of the ATPase Function

To verify the functionality of the Hsp90 protein, both disperse and bound to the particles and its capability to undergo ATP-dependent cyclical structural changes, a commercially available quantitative ATP detection kit was utilized (abcam luminescent ATP detection assay kit, ref. ab113849). This kit contains all the necessary components, including magnesium (Mg), for assessing ATP hydrolysis by both free Hsp90 particles and NP@Hsp90 particles. The assay operates on the principle of the luciferase–luciferin reaction. As ATP is consumed by the produced ATPase, the bioluminescence decreases, resulting in a decay in light intensity. The measured decrease in light intensity (Figure 1f) is directly proportional to the amount of ATP that has been hydrolyzed and is typically expressed in relative light units (RLU). Naked particles were used as negative controls.

### Aggregation Assay

Aggregation assays in Figure 2 were performed with in-house produced and purified α-synuclein mutant E57K protein (6xHis:α-Syn-E57K), incubated under amyloidogenic conditions (72 h at 37 °C in rotation). Raman spectroscopy studies were performed on the total extract of α-Syn-E57K overexpressing bacteria after sonication and disruption, centrifugation at 20,000*g*, and filtering through a 0.22 pore filter. The final sample was diluted in Na_2_HPO_4_ buffer to a final concentration of 50 mM. The estimated total protein amounts in the assays were calculated based on semi-quantitative measurements of the samples on acrylamide gels. The approximate quantities were determined to be around 200 μg of α-Syn-E57K, approximately 40 μg of free Hsp90, and approximately 20 μg of Hsp90 bonded to nanoparticles.

### Raman Spectroscopy

Raman spectroscopic measurements on the samples were performed with the 488 nm excitation laser line in an air-ambient environment using a Horiba T64000 Confocal Raman system equipped with a nitrogen-cooled CCD detector. The excitation laser line power was kept below 1 mW to avoid the sample heating effect. The Raman signal was collected by an Olympus 100× objective (N.A. = 0.9) with a spectral resolution of 0.6 cm^–1^. The intensity of the amide I β-sheet band (1665 cm^–1^) was normalized to the intensity of the deformation CH_3_–CH_2_ band (1451 cm^–1^).

## Supporting Information Available

Normalized values of the intensity of amide I peaks in arbitrary units and diagram of the sequences of the genetically engineered proteins designed (PDF)
