# Supplementary material for: Enhanced Inhibition of Amyloid Formation by Heat Shock Protein 90 Immobilized on Nanoparticles
Source: ACS Chem Neurosci. 2023 Jul 20;14(15):2811–7. doi: 10.1021/acschemneuro.3c00370 (PMC10401628; doi:10.1021/acschemneuro.3c00370)
Supplement: Supplementary file 1 — cn3c00370_si_001.pdf [file cn3c00370_si_001.pdf]

Supplementary Information

Enhanced inhibition of amyloid formation by heat shock protein 90 immobilized on nanoparticles.

Ana Rodríguez-Ramos, Jesús A. González, Mónica L. Fanarraga*.
Grupo de Nanomedicina. Universidad de Cantabria, Instituto Valdecilla - IDIVAL, Avda. Herrera Oria s/n, 39011, Santander, Spain.

Supporting table:

	Control - SNCA	SNCA + Hsp90	SNCA + PS@Hsp90	SNCA + SiO2@Hsp90
Measurement 1	1.408	0.55	0.45	0.34
Measurement 2	1.24	0.52	0.4	0.31
Measurement 3	1.32	0.59	0.48	0.37

Table S1. Normalized values of the intensity of Amide I peaks in arbitrary units.

Supporting figure:

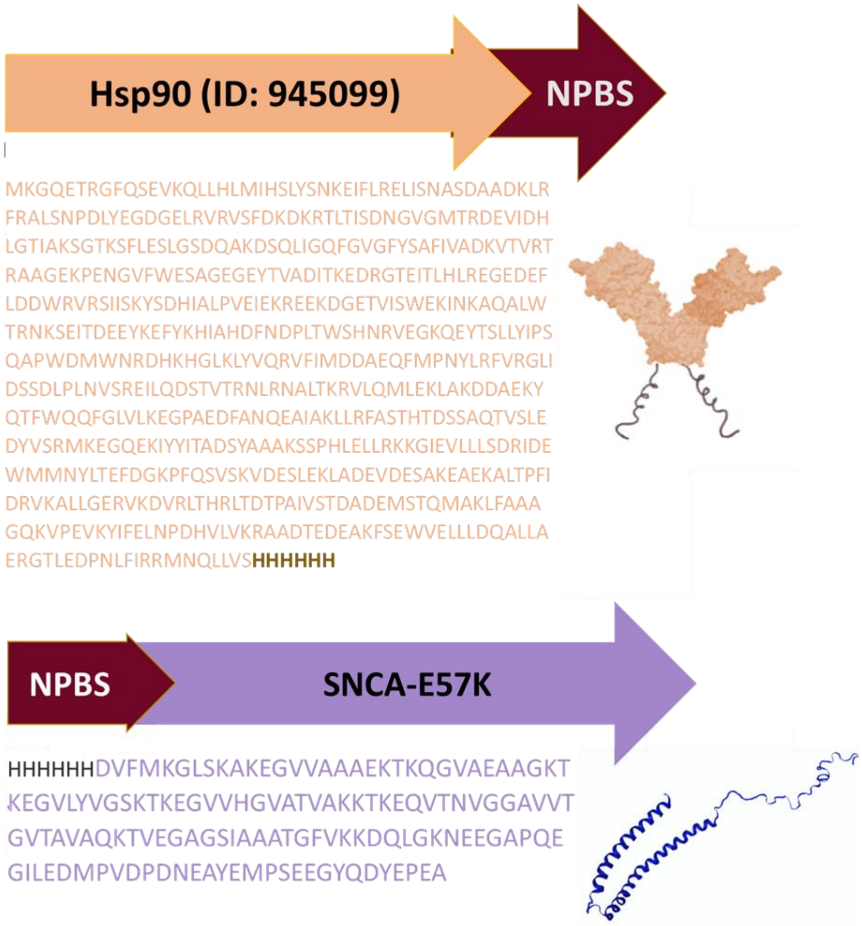

Figure S1. (top) Diagram of the sequences of the genetically engineered Hsp90 containing the nanoparticle binding site (NPBS), and the human α-Syn E57K mutant used.
